# Original TDAE Strategy Using Propargylic Chloride: Rapid Access to 1,4-Diarylbut-3-ynol Derivatives

**DOI:** 10.3390/molecules18021540

**Published:** 2013-01-25

**Authors:** Manon Roche, Thierry Terme, Patrice Vanelle

**Affiliations:** Laboratoire de Pharmaco-Chimie Radicalaire, Faculté de Pharmacie, Institut de Chimie Radicalaire ICR, UMR 7273, Aix-Marseille Univ, CNRS, 27 Boulevard Jean Moulin, 13385 Marseille Cedex 05, France

**Keywords:** TDAE, propargylic alcohol, organic reducing agent, aromatic aldehyde

## Abstract

We report herein the first synthesis of propargylic alcohols using an organic reducing agent. Diarylbutynol derivatives are formed in moderate to good yields under mild conditions from the reaction of 1-(3-chloroprop-1-ynyl)-4-nitrobenzene with various aromatic aldehydes using tetrakis(dimethylamino)ethylene (TDAE) as reductant.

## 1. Introduction

**Figure 1:**
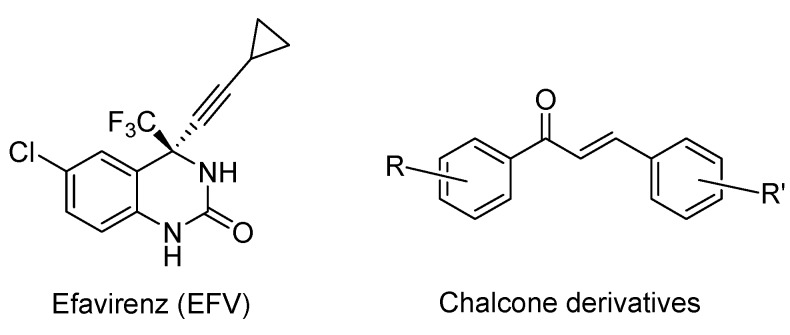
Example of molecules synthesized via propargylic alcohol derivatives.

Propargylic alcohol derivatives have a major role to play in medicinal chemistry. They can act as intermediates for many complex organic molecules with valuable biological activities [1,2], for example efavirenz (Figure 1), an antiretroviral agent. Chalcone scaffolds (Figure 1), prepared from propargylic alcohols such as diaryl butynols, have been already used in the design and synthesis of molecules with anticancer activities. They are particularly important in the synthesis of novel antitubulin agents [3,4,5].

Different methods can be used to obtain propargylic alcohols. The most commonly used method is the addition of an acetylenic derivative to carbonyl compounds mediated by titanium, dialkyl zinc, *etc.* [2]. Tetrakis(dimethylamino)ethylene (TDAE) is an organic reducing agent [6,7] which reacts with haloalkyl derivatives to generate an anion under mild conditions via two sequential transfers of one electron. TDAE methodology has been widely explored in fluorine chemistry [8]. Since 2002, we have been applying this methodology in the nitrobenzylic series with various electrophiles such as aromatic aldehydes [9,10,11,12], ketones [9,10,11,12], α-ketoesters [13,14,15], diethyl ketomalonate [13,14,15], α-ketolactams [16], α-diketones [17,18] and sulfonimine derivatives [19]. In this way, we have developed a program to explore the overall potential of TDAE-based chemistry in a medicinal setting [9,12,19,20,21]. To date, there has been no example of a TDAE-initiated reaction on alkyne derivatives in order to synthesize propargylic alcohols.

In a recent work, we explored the concept of Long-Distance-S_RN_1 (LD-S_RN_1) on a propargylic chloride derivative such as 1-(3-chloroprop-1-ynyl)-4-nitrobenzene [22]. This latter compound also constitutes a potential substrate for the preparation of a propargylic anion using the TDAE strategy. In continuation of our research program directed towards the development of original synthetic methods in medicinal chemistry [23,24,25,26,27,28], we examined the use of TDAE methodology in the alkyne series. We report herein the synthesis of diarylbutynol derivatives from the reaction of 1-(3-chloroprop-1-ynyl)-4-nitrobenzene with various aromatic aldehydes in the presence of TDAE.

## 2. Results and Discussion

**Scheme 1:**
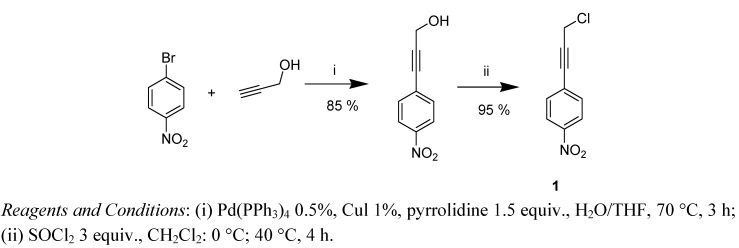
Synthesis of chloride **1**.

The synthesis of 1-(3-chloroprop-1-ynyl)-4-nitrobenzene (**1**) was inspired by Chinchilla’s work [29] on Sonogashira reactions and was performed as previously described (Scheme 1) [22].

**Scheme 2:**
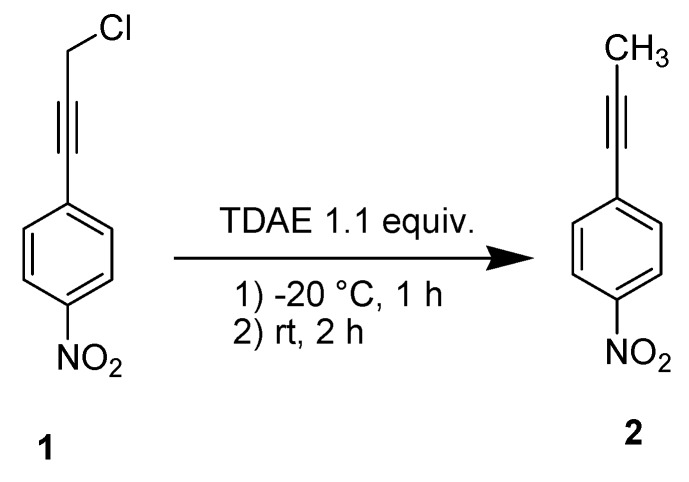
TDAE-initiated reactivity of chloride **1**.

In order to evaluate the reducibility of the substrate **1**, we treated it under classical TDAE conditions [9] but without electrophile. This reaction led to the reduction product **2** (Scheme 2) in 90% yield, and confirmed the reducibility of **1** by TDAE.

**Scheme 3:**
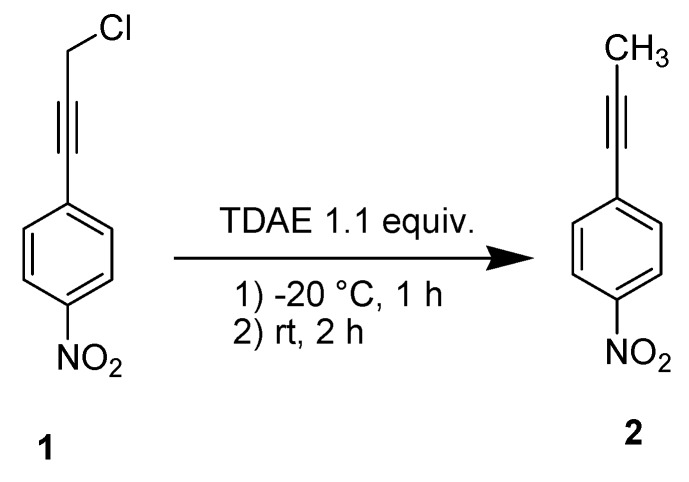
TDAE-initiated reactivity of chloride **1** and *p*-chlorobenzaldehyde **3a**.

We therefore proceeded to explore whether of a propargylic anion could be formed using TDAE with nucleophilic addition to a carbonyl derivative as electrophile. The first attempt was made under classical TDAE conditions [9]. The reaction of the 1-(3-chloroprop-1-ynyl)-4-nitrobenzene (**1**) with three equiv. of *p*-chlorobenzaldehyde (**3a**) in the presence of 1.1 equiv. of TDAE at −20 °C for 1 h (stage 1) followed by 2 h at rt (stage 2) yielded diarylbutynol **4a** in 40% yield (Entry 1, Table 1). Aromatic aldehydes were chosen as electrophiles based on our previous TDAE studies (more reactive electrophiles for the TDAE strategy) and with the view to obtaining chalcone precursors. In order to optimize this reaction, we changed some parameters (solvent, amount of aldehyde, solvent volume, temperature during stage 1 or stage 2 and reaction time of stage 2), as shown in Table 1 and Scheme 3. During this study, the formation of an oxirane derivative **5a** was observed as by-product when we used 10 or 20 mL of DMF (Entries 4 and 7).

Modification of certain parameters (solvent, aldehyde amount and reaction time) did not lead to any increase in yield of **4a**. Reducing the temperature of stage 1 (−20 °C to −50 °C; Entry 8) did not change diarylbutynol yields, but did improve the treatment of the reaction. Recently, we showed that the temperature of stage 2 affects TDAE reactivity in the *o*-nitrobenzylic series [30]. Thus, in Entry 9 the reaction was stirred under 60 °C for 2 h in stage 2 and diarylbutynol **4a** was obtained in 65% yield. As observed in benzylic [9,13], heterocyclic [14] and quinonic [12] series, the formation of this product may be explained by an ionic addition of carbanion, formed by action of TDAE with 1-(3-chloroprop-1-ynyl)-4-nitrobenzene, on the carbonyl group of the benzaldehyde.

**Table 1:** Reactivity of 1-(3-chloroprop-1-ynyl)-4-nitrobenzene **1** with *p*-chlorobenzaldehyde **3a**.

Entry ^a^	Equiv. of 3a	Solvent	T_1_^d^	Time of reaction (stage 2)	T_2_^e^	(%) Yield ^c^		
						1	4a	5a
1	3	DMF, 4 mL	−20 °C	2 h	rt	-	40	-
2 ^b^	3	THF, 4 mL	−20 °C	2 h	rt	-	-	-
3	3	MeCN, 4 mL	−20 °C	2 h	rt	85	-	-
4	3	DMF, 4 mL	−20 °C	6 h	rt	-	6	traces
5	1	DMF, 4 mL	−20 °C	2 h	rt	-	12	-
6	4	DMF, 4 mL	−20 °C	2 h	rt	-	20	-
7	3	DMF, 10 mL	−20 °C	2 h	rt	-	38	20
8	3	DMF, 4 mL	−50 °C	2 h	rt	-	45	-
**9**	**3**	**DMF, 4 mL**	**−50 °C**	**2 h**	**60 °C**	**-**	**65**	**-**
10	3	DMF, 10 mL	−50 °C	2 h	rt	-	25	35
11	3	DMF, 20 mL	−20 °C	2 h	rt	-	30	traces
12	3	DMF, 20 mL	−50 °C	2 h	rt	-	38	25
13	3	DMF, 20 mL	−50 °C	6 h	rt	-	25	25

^a^ All reactions were performed using strictly anhydrous solvent, with 1 equiv. (0.5 mmol) of chloride **1**, 1.1 equiv. of TDAE and 1 h for stage 1; ^b^ Only degradation products were observed; ^c^ All yields refer to the chromatographically isolated products and are relative to chloride **1**; ^d^ T_1_: Temperature stage 1; ^e^ T_2_: Temperature stage 2.

**Scheme 4:**
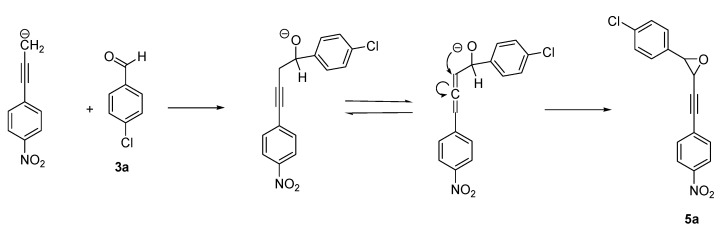
Hypothetical pathway for the formation of oxirane derivative.

Otherwise, the reaction led to oxirane **5a** as by-product in entries 4 and 7. Using the TDAE methodology, the formation of oxirane derivatives with *gem*-dihalomethyl derivatives has already been described [11], but not with monohalogenated compounds. To elucidate the pathway for the formation of this product, certain parameters were modified (Table 1, Entries 10–13). In these assays, the best yield (35%) of product **5a** was obtained for Entry 10, in the presence of 1.1 equiv. of TDAE at −50 °C for 1 h (stage 1) followed by 2 h at rt (stage 2), in 10 mL of extra-dry DMF. The reaction with the highest volume of DMF (Entry 12) did not lead to an increased yield of product **5a**. All this suggests the formation of oxirane **5a** in dilute solutions by an intramolecular rearrangement via the propargylic/allenic equilibrium (Scheme 4). ^1^H-NMR spectral studies identify oxirane as *cis*-isomer by their coupling constant. Two distinct doublets appeared in the region at 3.97–4.25 ppm with a coupling constant *J* = 3.8 Hz, in agreement with the literature [31,32].

**Scheme 5:**
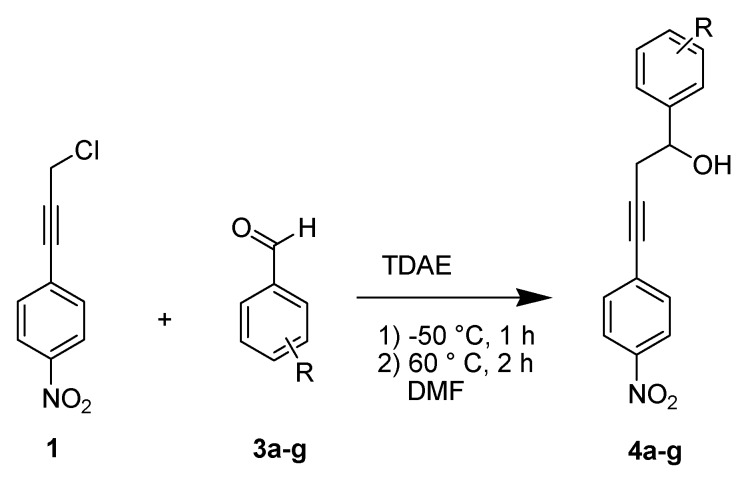
Extension of the reactivity of chloride **1** with various benzaldehydes **3a**–**g**.

In order to generalize and to confirm this new and original methodology of synthesizing diarylbutynol derivatives, we extended this study to other aromatic aldehydes using an optimized protocol. The reaction of 1-(3-chloroprop-1-ynyl)-4-nitrobenzene **1** with electrophiles **3a**–**g** in the presence of TDAE, under the conditions of Entry 9 (Table 1), led to the corresponding diarylbutynol derivatives **4a**–**g** in moderate to good yields (30% to 65%) as shown in Table 2 and Scheme 5.

**Table 2:** Reaction of chloride **1** with various aromatic aldehydes ^a^.

Aromatic aldehyde	R		(%) Yield ^b^
4-Chlorobenzaldehyde	4-Cl	**4a**	65
4-Nitrobenzaldehyde	4-NO_2_	**4b**	57
4-Fluorobenzaldehyde	4-F	**4c**	34 ^c^
4-Tolualdehyde	4-CH_3_	**4d**	traces
4-Trifluoromethylbenzaldehyde	4-CF_3_	**4e**	51
2-Nitro-4,5-dimethoxybenzaldehyde	2-NO_2_-4,5-(OCH_3_)_2_	**4f**	32
Benzaldehyde	H	**4g**	30 ^d^

^a^ All reactions are performed using 3 equiv. of aromatic aldehyde **3a**–**g**, 1 equiv. (0.5 mmol) of chloride **1**, 1.1 equiv. of TDAE in 4 mL of strictly anhydrous DMF, stirred at −50 °C for 1 h and then warmed at 60 °C for 2 h; ^b^ All yields refer to the chromatographically isolated products and are relative to chloride **1**; ^c^ We observed the formation of reduction product **2** in 25% yield; ^d^ We observed the formation of reduction product **2** in 20% yield.

As already described in the nitrobenzylic [9,10] or quinonic [12] series, aromatic aldehydes with an electron withdrawing group are more reactive than aromatic aldehydes substituted by an electron-donor group such as methyl (compound **3d**). The formation of the reduction product **2**, isolated as by-product with 4-tolualdehyde (**3d**) or benzaldehyde (**3g**), has already been detected in our previous studies when the substrate is more reducible and the electrophile is less reactive [15,33,34].

## 3. Experimental

### 3.1. General

Elemental analysis or mass spectrometry were performed by the Spectropole Centre, Aix Marseille University (Marseille, France) The ^1^H- and ^13^C-NMR spectra were determined on a Bruker AC 200 spectrometer operating at 200 and 50 MHz, respectively. The ^1^H-NMR chemical shifts are reported as parts per million downfield from tetramethylsilane (Me_4_Si), the ^13^C chemical shifts were referenced to the solvent peaks [CDCl_3_: 76.9 ppm and (CD_3_)_2_CO: 206.3 ppm and 29.8 ppm]. Absorptions are reported with the following notations: s, singlet, d, doublet, t, triplet, q, quartet, m, a more complex multiplet or overlapping multiplets. The following absorbents were used for column chromatography: silica gel 60 (Merck, particle size 0.0063–0.200, 70–230 mesh ASTM). TLC was performed on 5 cm × 10 cm aluminium plates coated with silica gel 60 F-254 (Merck) with an appropriate eluent.

### 3.2. General Procedure for TDAE Reaction with Aromatic Aldehydes

All materials were dried for one day at 120 °C. Into a two-necked flask equipped with a silica-gel drying tube and a nitrogen inlet, was added, under nitrogen at –50 °C, an anhydrous DMF solution (4 mL) of **1** (0.1 g, 0.51 mmol) and aldehyde **3a**–**g** (1.53 mmol, 3 equiv.). The solution was stirred and maintained at this temperature for 30 min and then the TDAE (0.127 mL, 0.55 mmol, 1.1 equiv.) was added dropwise (via a syringe). A green color immediately developed. The solution was vigorously stirred at −50 °C for 1 h and then warmed at 60 °C for 2 h. After this time TLC analysis (dichloromethane) clearly showed that **1** was totally consumed. The brown turbid solution was hydrolyzed with H_2_O (5 mL). The aqueous solution was extracted with dichloromethane (3 ° 10 mL), the combined organic layers washed with brine (3 × 10 mL) and dried over MgSO_4_. Evaporation of the solvent left the crude product as a viscous orange liquid. Purification by silica gel chromatography (eluent dichloromethane) gave the corresponding diarylbutynols **4a**–**g**.

*1-(4-Chlorophenyl)-4-(4-nitrophenyl)but-3-yn-1-ol* (**4a**). A yellow oil (98 mg, 65%). ^1^H-NMR (CDCl_3_): δ 8.16 (2H, d, *J* = 8.8 Hz), 7.50 (2H, d, *J* = 8.8 Hz), 7.37 (4H, s), 4.98 (1H, t, *J* = 6.2 Hz), 2.90 (2H, d, *J* = 6.2 Hz). ^13^C-NMR (CDCl_3_): δ 146.9 (C), 140.9 (C), 133.9 (C), 132.4 (2CH), 130.1 (C), 128.7 (2CH), 127.2 (2CH), 123.5 (2CH), 91.6 (C), 81.7 (C), 71.8 (CH), 30.5 (CH_2_). HRMS calcd. for C_16_H_12_ClNO_3_: [M+H]^+^ = 302.0578, found: [M+H]^+^ = 302.0577.

*1,4-Bis(4-nitrophenyl)but-3-yn-1-ol* (**4b**). A yellow solid (90 mg, 57%). ^1^H-NMR [(CD_3_)_2_CO]: δ 8.25 (2H, d, *J* = 8.6 Hz), 8.19 (2H, d, *J* = 8.6 Hz), 7.83 (2H, d, *J* = 8.6 Hz), 7.59 (2H, d, *J* = 8.6 Hz), 5.19 (1H, m), 2.97 (2H, d, *J* = 5.5 Hz). ^13^C-NMR [(CD_3_)_2_CO]: δ 152.5 (C), 148.3 (C), 147.9 (C), 133.2 (2CH), 131.3 (C), 128.1 (2CH), 124.4 (2CH), 124.0 (2CH), 93.2 (C), 82.0 (C), 71.7 (CH), 30.6 (CH_2_). HRMS calcd. for C_16_H_12_N_2_O_5_: [M+Na]^+^ = 335.0638, found: [M+Na]^+^ = 335.0638.

*1-(4-Fluorophenyl)-4-(4-nitrophenyl)but-3-yn-1-ol* (**4c**). A yellow oil (50 mg, 34%). ^1^H-NMR (CDCl_3_): δ 8.15 (2H, d, *J* = 8.8 Hz), 7.5 (2H, d, *J* = 8.8 Hz), 7.40 (2H, m), 7.08 (2H, m), 4.98 (1H, t, *J* = 6.3 Hz), 2.90 (2H, d, *J* = 6.3 Hz). ^13^C-NMR (CDCl_3_): δ 162.3 (1C, d, *J* = 246.6 Hz), 147.0 (C), 138.2 (C), 132.4 (2CH), 130.2 (C), 127.6 (2CH, d, *J* = 8.4 Hz), 123.6 (2CH), 115.4 (2CH, d, *J* = 21.6 Hz), 91.6 (C), 81.7 (C), 71.9 (CH), 30.6 (CH_2_). HRMS calcd. for C_16_H_12_FNO_3_: [M+Na]^+^ = 308.0693, found: [M+Na]^+^ = 308.0692.

*4-(4-Nitrophenyl)-1-p-tolylbut-3-yn-1-ol* (**4d**). Traces. ^1^H-NMR (CDCl_3_): δ 8.29 (2H, d, *J* = 8.8 Hz), 7.40 (2H, m), 7.10 (4H, m), 7.08 (2H, m), 4.98 (1H, m), 2.41 (3H, s).

*4-(4-Nitrophenyl)-1-[4-(trifluoromethyl)phenyl]but-3-yn-1-ol* (**4e**). A yellow oil (87 mg, 51%). ^1^H-NMR (CDCl_3_): δ 8.17 (2H, d, *J* = 8.8 Hz), 7.60 (4H, m), 7.50 (2H, d, *J* = 8.8 Hz), 5.06 (1H, m), 2.93 (2H, d, *J* = 6.3 Hz), 2.42 (1H, bs, OH). ^13^C-NMR (CDCl_3_): δ 147.0 (C), 146.3 (C), 132.4 (2CH), 130.3 (1C, q,*J* = 32.3 Hz), 129.9 (C), 126.1 (2CH), 127.5 (2CH, q,*J* = 3.6 Hz), 124.0 (C, q,*J* = 272.3 Hz), 123.6 (2CH), 91.2 (C), 81.9 (C), 71.9 (CH), 30.5 (CH_2_). HRMS calcd. for C_17_H_12_F_3_NO_3_: [M+Na]^+^ = 358.0661, found: [M+Na]^+^ = 358.0661.

*1-(4,5-Dimethoxy-2-nitrophenyl)-4-(4-nitrophenyl)but-3-yn-1-ol* (**4f**). A brown oil (60 mg, 32%). ^1^H-NMR (CDCl_3_): δ 8.15 (2H, d, *J* = 8.8 Hz), 7.62 (1H, s), 7.51 (2H, d, *J* = 8.8 Hz), 7.40 (1H, s), 5.76 (1H, dd, *J* = 6.9 and 4.3 Hz), 3.96 (6H, s), 3.15 (1H, dd, *J* = 16.9 and 4.3 Hz), 3.05 (1H, dd, *J* = 16.9 and 6.9 Hz). ^13^C-NMR (CDCl_3_): δ 153.7 (C), 148.2 (C), 146.9 (C), 139.7 (C), 133.4 (C), 132.4 (2CH), 130.2 (C), 123.6 (2CH), 109.2 (CH), 107.8 (CH), 91.7 (C), 81.8 (C), 67.7 (CH), 56.4 (2OCH_3_), 29.7 (CH_2_). HRMS calcd. for C_18_H_16_N_2_O_7_: [M+Na]^+^ = 395.0850, found: [M+Na]^+^ = 395.0849.

*4-(4-Nitrophenyl)-1-phenylbut-3-yn-1-ol* (**4g**). A yellow oil (41 mg, 30%). ^1^H-NMR (CDCl_3_): δ 8.15 (2H, d, *J* = 8.8 Hz), 7.50 (2H, d, *J* = 8.8 Hz), 7.40 (5H, m), 5.00 (1H, t, *J* = 6.3 Hz), 2.92 (2H, d, *J* = 6.3 Hz). ^13^C-NMR (CDCl_3_): δ 146.9 (C), 142.5 (C), 132.4 (2CH), 130.3 (C), 128.6 (2CH), 128.2 (CH), 125.8 (2CH), 123.5 (2CH), 92.2 (C), 81.5 (C), 72.6 (CH), 30.5 (CH_2_). HRMS calcd. for C_16_H_13_NO_3_: [M+NH_4_]^+^ = 285.1234, found: [M+NH_4_]^+^ = 285.1238.

Compound **5a** was isolated in 35% yield according to the general procedure cited above, but using 10 mL of DMF as solvent, rt for stage 2 (Table 1, Entry 10) and purification by silica gel chromatography (dichloromethane/petroleum ether 7/3).

*2-(4-Chlorophenyl)-3-[(4-nitrophenyl)ethynyl]oxirane (cis isomer)* (**5a**). A yellow oil (57 mg, 35%). ^1^H-NMR (CDCl_3_): δ 8.14 (2H, d, *J* = 8.8 Hz), 7.40 (2H, d, *J* = 8.8 Hz), 7.39 (4H, s), 4.24 (1H, d, *J* = 3.8 Hz), 3.98 (1H, d, *J* = 3.8 Hz). ^13^C-NMR (CDCl_3_): δ 134.6 (C), 132.6 (2CH), 132.5 (C), 128.5 (C), 128.2 (4CH), 123.6 (2CH), 88.6 (C), 84.1 (C), 58.7 (CH), 48.4 (CH). C–NO_2_ was not observed under these experimental conditions. HRMS calcd. for C_16_H_10_ClNO_3_: [M+NH_4_]^+^ = 317.0687, found: [M+NH_4_]^+^ = 317.0687.

## 4. Conclusions

This study is the first to report on the use of the TDAE methodology in an alkyne series. The reaction of 1-(3-chloroprop-1-ynyl)-4-nitrobenzene (**1**) with aromatic aldehydes led to the corresponding alcohols **4a**–**g** in moderate to good yields. We present here the first example of the formation of a propargylic carbanion via the TDAE methodology. This is an original and mild method to synthesize diarylbutynol derivatives as chalcone precursors with potential for pharmacological use as anticancer agents.
